# Supplementary material for: ENaC-mediated sodium influx exacerbates NLRP3-dependent inflammation in cystic fibrosis
Source: eLife. 2019 Sep 18;8:e49248. doi: 10.7554/eLife.49248 (PMC6764826; doi:10.7554/eLife.49248)
Supplement: Supplementary file 1. — Patients with Cystic Fibrosis (CF), systemic autoinflammatory diseases (SAID), non-CF bronchiectasis (NCFB) and healthy controls (HC). All CF donors were F508del/F508del homozygous (n = 30) with no sign of infection. Three of the patients with NCFB had primary ciliary dyskinesia (PCD) and one patient with NCFB had an unknown genotype. All patients with a SAID had characterised mutations in a known disease-causing gene (Tumor Necrosis Factor Receptor Associated Periodic Syndrome (TRAPS) n = 2, Muckle-Wells n = 2, A20 haploinsufficiency n = 1, Pyrin-Associated Autoinflammation with Neutrophilic Dermatosis (PAAND) n = 1, Familial Mediterranean Fever (FMF) n = 2, Hyper IgD Syndrome (HIDS) n = 2 and Schnitzler syndrome n = 1). BMI: Body Mass Index; FEV: Forced expiratory volume; CRP: C-reactive protein. [file elife-49248-supp1.pptx]

## Slide 1
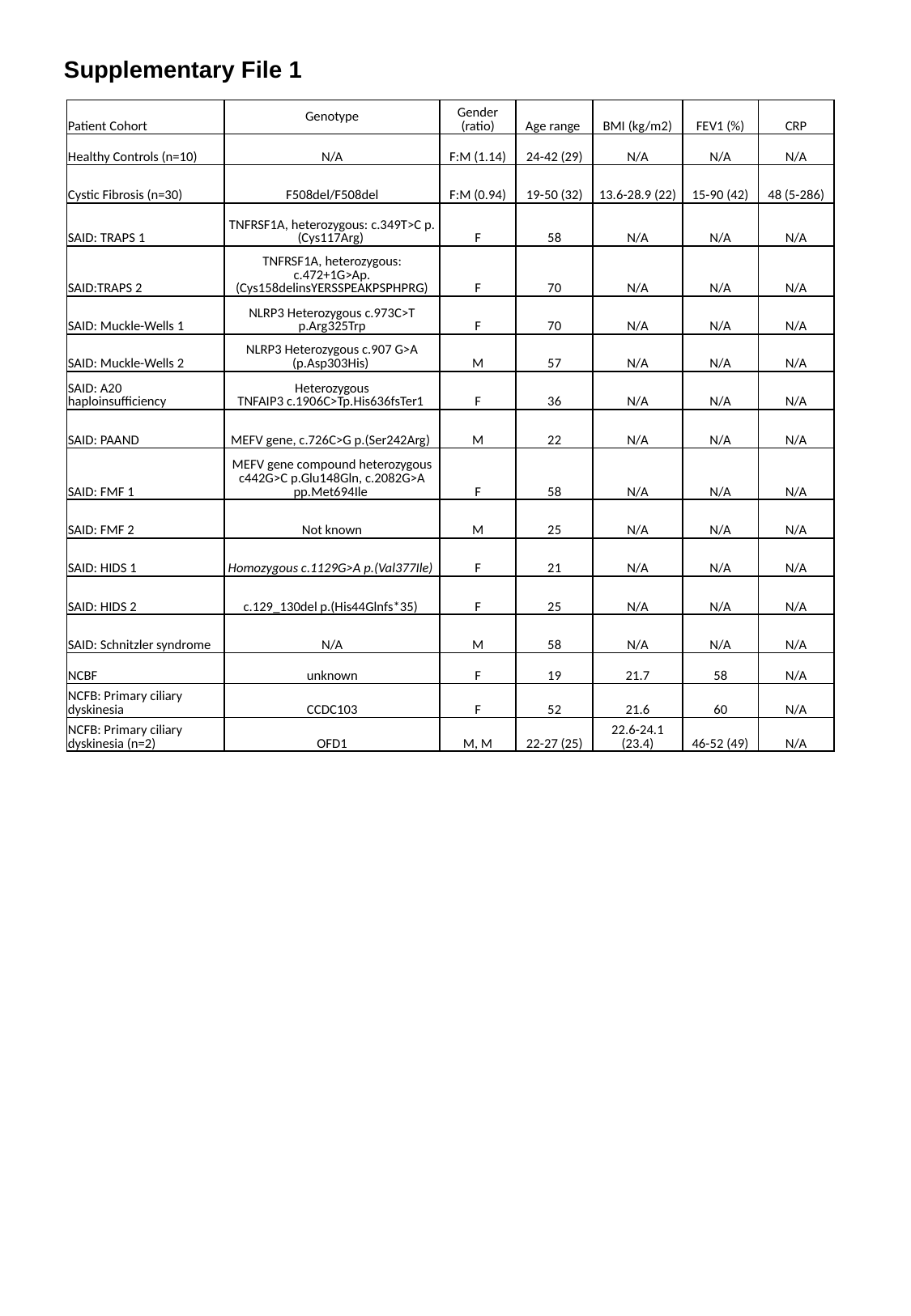

Supplementary File 1
| Patient Cohort | Genotype | Gender (ratio) | Age range | BMI (kg/m2) | FEV1 (%) | CRP |
| --- | --- | --- | --- | --- | --- | --- |
| Healthy Controls (n=10) | N/A | F:M (1.14) | 24-42 (29) | N/A | N/A | N/A |
| Cystic Fibrosis (n=30) | F508del/F508del | F:M (0.94) | 19-50 (32) | 13.6-28.9 (22) | 15-90 (42) | 48 (5-286) |
| SAID: TRAPS 1 | TNFRSF1A, heterozygous: c.349T>C p.(Cys117Arg) | F | 58 | N/A | N/A | N/A |
| SAID:TRAPS 2 | TNFRSF1A, heterozygous: c.472+1G>Ap. (Cys158delinsYERSSPEAKPSPHPRG) | F | 70 | N/A | N/A | N/A |
| SAID: Muckle-Wells 1 | NLRP3 Heterozygous c.973C>T p.Arg325Trp | F | 70 | N/A | N/A | N/A |
| SAID: Muckle-Wells 2 | NLRP3 Heterozygous c.907 G>A (p.Asp303His) | M | 57 | N/A | N/A | N/A |
| SAID: A20 haploinsufficiency | Heterozygous TNFAIP3 c.1906C>Tp.His636fsTer1 | F | 36 | N/A | N/A | N/A |
| SAID: PAAND | MEFV gene, c.726C>G p.(Ser242Arg) | M | 22 | N/A | N/A | N/A |
| SAID: FMF 1 | MEFV gene compound heterozygous c442G>C p.Glu148Gln, c.2082G>A pp.Met694Ile | F | 58 | N/A | N/A | N/A |
| SAID: FMF 2 | Not known | M | 25 | N/A | N/A | N/A |
| SAID: HIDS 1 | Homozygous c.1129G>A p.(Val377Ile) | F | 21 | N/A | N/A | N/A |
| SAID: HIDS 2 | c.129\_130del p.(His44Glnfs\*35) | F | 25 | N/A | N/A | N/A |
| SAID: Schnitzler syndrome | N/A | M | 58 | N/A | N/A | N/A |
| NCBF | unknown | F | 19 | 21.7 | 58 | N/A |
| NCFB: Primary ciliary dyskinesia | CCDC103 | F | 52 | 21.6 | 60 | N/A |
| NCFB: Primary ciliary dyskinesia (n=2) | OFD1 | M, M | 22-27 (25) | 22.6-24.1 (23.4) | 46-52 (49) | N/A |
